# An analysis of intra array repeats: the good, the bad and the non informative

**DOI:** 10.1186/1471-2164-7-136

**Published:** 2006-06-05

**Authors:** Yedid Elbez, Shlomit Farkash-Amar, Itamar Simon

**Affiliations:** 1 Faculté des Sciences de la vie., Université Louis Pasteur 28 rue Goethe., 67083 Strasbourg cedex, France; 2 Department of Molecular Biology, Hebrew University – Hadassah Medical School, Jerusalem 91120, Israel

## Abstract

**Background:**

On most common microarray platforms many genes are represented by multiple probes. Although this is quite common no one has systematically explored the concordance between probes mapped to the same gene.

**Results:**

Here we present an analysis of all the cases of multiple probe sets measuring the same gene on the Affymetrix U133a GeneChip and found that although in the majority of cases both measurements tend to agree there are a significant number of cases in which the two measurements differ from each other. In these cases the measurements can not be simply averaged but rather should be handled individually.

**Conclusion:**

Our analysis allows us to provide a comprehensive list of the correlation between all pairs of probe sets that are mapped to the same gene and thus allows microarray users to sort out the cases that deserve further analysis. Comparison between the set of highly correlated pairs and the set of pairs that tend to differ from each other reveals potential factors that may affect it.

## Background

High density DNA microarrays are extensively used by biologists in order to gain information about the expression levels of thousands of genes simultaneously. The advantage of the genomic approach is that it allows the study of cell behavior on the systems level rather than on the gene specific level [1]. One of the hurdles of this approach is that the confidence one has in each measurement is lower in comparison to the confidence gained by classical molecular biology techniques[2], thus extensive statistical analysis is needed in order to define the significance of the results. One of the approaches used to asses the quality of results is comparison between replicates [3]. This is accomplished by both printing each DNA fragment several times on the array (internal replicates) and repeating the experiment multiple times (experimental replicates). While internal replicates usually give very similar results, experimental replicates may differ from one another due to variation in the biological samples, exact experimental conditions and array processing [4]. There is yet another type of replicates – multiple array spots that are mapped to different regions of the same gene, thus allowing multiple measurements of the RNA levels in the exact same biological sample on the same array. We call these "intra array repeats" and not "intra array replicates" in order to highlight the fact that they are not identical. Although this is quite common, for example 35% of the genes on Affymetrix U133a array are represented by multiple probe sets, no systematic evaluation of the concordance between multiple representations of the same gene has been reported, thus leaving microarray users without clear guidelines how to deal with this problem.

A related issue which has been studied extensively in recent years is the question of cross platform comparisons in which researchers try to compare results obtained for the same gene on different microarray platforms. Overall there is only limited agreement between experiments performed on different platforms (reviewed by [5,6]). Many factors can contribute to this cross platform inconsistency, including differences in RNA processing, noise levels [7], the statistical analysis required by each platform [8] and the sequences chosen to represent the gene by the different manufacturers [9,10]. Recent studies suggest that the latter issue is very important since there is much more agreement between platforms when only spots with overlapping sequences are used [11-13]. In contrast to cross platform comparisons which are affected by many variables, intra platform comparisons may be affected only by the sequences chosen to represent the gene and thus may serve as an excellent test case for assessing the quality of microarrays results. In this study we identified all groups of probe sets mapped to the same gene that are present on the Affymetrix U133a GeneChip and examined the degree of agreement between those pairs. In most cases we observed significant correlations between the pairs suggesting that both probe sets are reporting similar results. However, we did find a large subset that differs significantly from one another. The identification of these pairs is important for analyzing expression profile results, since in these cases the investigator can not rely on the expression levels reported by those probes.

## Results

### Gene expression data

**Figure 1:**
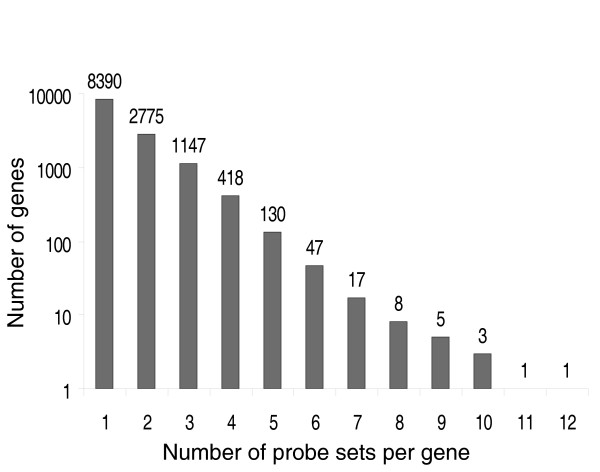
**GeneChip U133a properties**. Histogram showing the number of probe sets per gene on the Affymetrix U133a array.

In order to compare RNA measurements from two different regions of a gene on the same array, we chose to explore the Affymetrix U133a platform since it is a very popular platform with thousands of publicly available datasets. The U133a platform contains 20,267 probe sets which are mapped to 12,942 distinct genes with 4,552 of them represented by multiple probe sets (figure 1). Overall this creates 11,722 pairs of probe sets in which both members are mapped to the same gene. For our analysis we chose four publicly available data sets each containing at least 29 experiments (table 1) and downloaded the data from the Gene Expression Omnibus database. Each of these datasets were acquired in different laboratories, using different cells and growth conditions, thus we analyzed each of them separately.

Since we are interested in comparing the changes in transcriptional level of both members of each pair of probe sets, we defined as informative only probe sets that showed changes in transcription along the different measurements in a dataset. Only those probe sets were included in our analysis (see methods).

### Correlation coefficient

**Figure 2:**
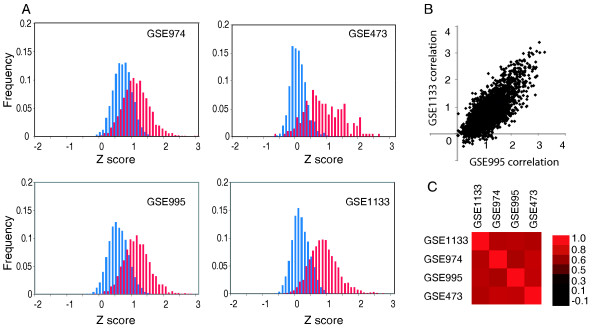
**Correlation between pairs of probe sets**. A. Histograms of the Fisher transformed Pearson correlation values (Z score) of related (red) and not related (blue) pairs of probe sets in the four datasets examined. Only informative pairs (see methods) are shown. B. Scatter plot showing the Fisher transformed Pearson correlation values of all the pairs in two datasets. C. Comparison between the correlation values obtained in all four datasets. The color in the intersection between two datasets depicts the correlation values. The correlation disappears in shuffled data.

In order to asses the overall agreement within pairs of probe sets, we decided to look at the Pearson correlation between the set of measurements for each of the probe sets in each dataset. We chose the Pearson correlation as our metric since it is not sensitive to differences in scaling and average expression levels but rather compares the degree of linear relationship between the two probe-sets which is a better indication for the overall similarity. We calculated the correlation coefficient of all the informative pairs in each dataset. Histograms of these correlations and correlations obtained by a random list of pairs are shown in figure 2a. The statistical significance of the deviation of the correlation of each pair from the underlying distribution was calculated, and the results are summarized in additional file 1.

Comparison of the correlation values obtained in the different datasets revealed a high degree of correlation between them (figure 2b,c), suggesting that the agreement between pairs of probe sets is characteristic of the probe sets and is not heavily dependent on the particular dataset from which the data was derived.

### Statistical assessment of the results

One of the main purposes of our analysis was to guide microarray users in cases of genes that are represented by multiple probe sets. The users would like to know when the multiple measurements can be combined and when they should be handled with caution. In order to achieve this goal, we wanted to define a list of pairs which show overall good agreement between them. This list should be based on the combination of the correlation values calculated in each dataset. Thus for each pair, we combined the individual P values obtained for each dataset in which it was informative, resulting in a single combined P value for each pair. A list of all the pairs along with their combined P values is included in additional file 1. This list includes 6536 pairs of probe sets that were informative at least in one of the data sets and 77% of them showed significant correlation (FDR = 0.01).

**Figure 3:**
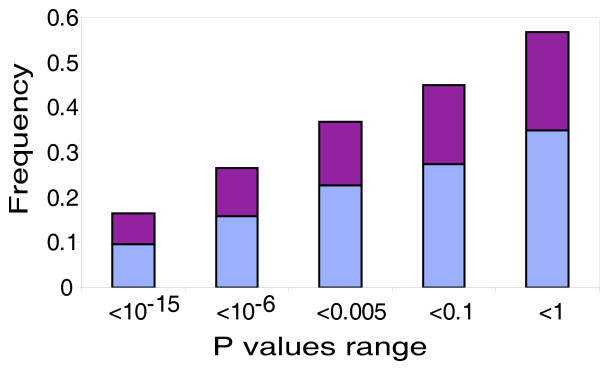
**Error rates after averaging pairs of probe sets**. The transcription values reported by pairs of probe sets was averaged and compared to the values obtained by each probe set alone. The frequency of cases in which the average wrongly reports changes in transcription of at least two fold (blue) or wrongly reports constant transcription (red) is shown to five exclusive ranges of P values.

Microarrays users frequently ask themselves what to do when a gene is represented by multiple probes. Our analysis provides a partial solution to this problem by mapping all the pairs of probe sets on the Affymetrix U133a platform and providing a significant measurement (P value) as to whether the transcription levels reported by the two different probe sets correlates. In order to demonstrate that the combined P value may be useful in analyzing microarray data, we calculated the number of cases in which averaging the intensity reported by a pair of probe sets is misleading. The clear dependency between the error rate level and the P value (figure 3) suggests that the P value is a useful guideline for dealing with intra array repeats.

### Exploring the "good" and "bad" pairs

Recent studies have shown that when performing cross platform comparisons between Affymetrix arrays and cDNA arrays, restricting the analysis to sequence-matched probes produces a higher level of consistency between the platforms [11-14]. These studies suggest that one of the main reasons for inconsistencies between different microarray platforms is the differences in the gene regions chosen to be printed for each platform. Different sequences may report different values for a variety of reasons that can be sorted into two categories – the two sequences are reporting the levels of different transcripts (due to annotation mistakes or different splice variants) alternatively, both sequences may report the transcription of the same transcript at a different accuracy (due to the well documented 3'-5' degradation of the mRNA or due to differences in hybridization efficiency of the two regions).

Intra array repeats are printed on the same array and therefore they share all the external conditions (including the biological sample, growth conditions, probe labeling and data handling) and differ from one another only in the particular sequence chosen to be printed on the array. Thus multiple probe sets printed on the same array serve as an excellent case for exploring the contribution of sequence effects to the overall deviation of the transcription values reported by each probe set. To this end we defined two large sub groups of pairs (each containing approximately 1500 pairs) – pairs that were highly correlated (P < 10^-15^; herein "good") and pairs which did not show a significant concordance between them (P > 0.1; herein "bad").

**Figure 4:**
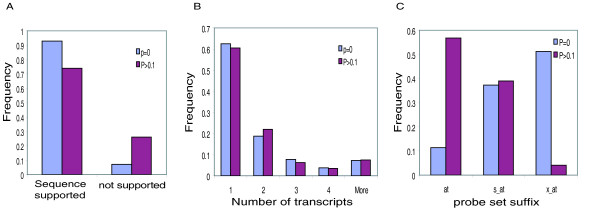
**Probe sets analysis**. Bar graphs showing the distribution of cases according to their (A) annotation quality; (B) average numbers of transcripts and (C) the Affymetrix probe set suffix in the "good" (red) and the "bad" (blue) pairs of probe sets. The results of a contingency table statistical test performed on each case revealed that the two distributions in B do not differ significantly from each other (P = 0.15, *X*^2 ^test) whereas in A and C the difference is significant (P < 10^-44^, Fisher Exact test and P = 10^-3^, *X*^2 ^test, respectively).

First we wanted to test the possibility that incorrect annotation contributes to the differences measured between pairs of probe sets. Recently, several reports claimed that Affymetrix annotation is not ideal and that using the sequences to reannotate the probe sets gives better results [15-17]. If mistakes in annotation were a major cause for the differences between pairs of probe sets we should expect to see a higher degree of misannotation in the "bad" pairs than in the "good" set of pairs. We calculated the percentage of cases in which Affymetrix gene assignment was supported by the GeneAnnot gene assignment. The results show that indeed misannotation has a big contribution to the disconcordance between the "bad" pairs, actually >25% of these pairs have no sequence support to the fact that they are reporting the transcript of the same gene (figure 4a).

Next we analyzed the affect of alternative splicing on probe accordance. If two probe sets are reporting the expression of two different splice variants of a gene, they may differ in the transcription values they are measuring. Thus another possible explanation for disagreement between pairs of probe sets is that each is reporting the transcription level of a different transcript of the same gene. In order to explore this idea, we looked at the number of known variant transcripts for each gene. Calculation of the average number of transcript variants in the "good" and in the "bad" pairs revealed that there are on average approximately 1.7 transcripts per gene and there is almost no difference in the distribution between the two groups (figure 4b). Moreover the frequency of the "bad" and of the "good" pairs is almost identical even in the group of genes with only a single known transcript, strongly suggesting that according to the current knowledge about alternative splicing, it can not be the reason for the deviation between pairs of probe sets in most cases.

Finally, we wanted to explore the contribution of cross hybridization between probes to the agreement between probe sets. To this end we used the Affymetrix suffix to the probe name as a measurement of the probe uniqueness. _at suffix designates a unique probe set, whereas _s_at and _x_at suffixes designate probe sets that can cross hybridize with multiple genes. To our surprise we found a very significant reverse correlation between the probe uniqueness and its agreement with another probe set. We found significant enrichment (P = 0.001) for the s_at and x_at suffixes in the "good" pairs (figure 4c), suggesting that cross hybridization between the genes is not the reason for the disagreement between probe sets.

## Discussion

Microarray users frequently encounter the problem of different values for the same gene resulting from multiple probe sets. Without clear guidelines from the manufacturers, each investigator deals with this problem in a different way, some are not aware of it and therefore either use the first appearance of the gene or use the probe set that fits their expectations better. Alternatively, users may use the average of all probe sets without validating that all indeed report similar transcription rates. In order to help microarray users to chose the right approach for multiple measurements of a gene in the Affymetrix U133a GeneChip we examined all such cases and developed a statistical method to asses the agreement between the measurements in each of these cases. Our method is based on the calculation of the Pearson correlation between all the measured values of a pair of probe sets along all the experiments in a data set. The Pearson correlation gives a good estimation of the overall agreement between such pairs without being sensitive to variability in the average expression level reported by the probe sets. We excluded from the analysis probes that were non informative either because their expression level was below detection or because their expression level did not change (>2 folds) along the experiments. As expected the correlation values obtained between probe sets from the same gene were much higher than those obtained from randomly chosen pairs of probe sets with 77% of the pairs reporting similar values. The pairs of probe sets where sorted according to the statistical significance of the Pearson correlation between them and a list of all the pairs together with their P value is provided [see additional file 2]. This list should serve as a guideline to the user: the lower the P value the higher chances that the two probes are reporting the same information whereas high P values suggest that the two observations should be treated individually (figure 3).

The division of the pairs of probe sets into those that tend to agree ("good") or disagree ("bad") allows us to examine the factors affecting it. The deviation between two probes that are mapped to the same gene may be due to several reasons including alternative splicing, mistakes in the annotation and differences in the efficiency each probe set reports the transcription. We found no evidence for the contribution of alternative splicing to the disagreement between probe sets (figure 4b). On the other hand by comparing the annotation in the sets of the "good" and the "bad" pairs we have shown that misannotation is obviously a main issue, with >25% of the "bad" pairs being misannotated (figure 4a) thus in cases of disagreement between probe sets one should first validate that both probe sets are indeed annotated correctly.

Recent studies about cross platform comparisons found that the best results are obtained if the comparison is done on the probe level rather than on the probe set level [12,18,19], suggesting that the actual sequence has a big contribution to the array results. In accordance with this idea we found that pairs of probe sets that share at least one common probe show almost always significant agreement (97.6% of 970 such cases passed the FDR of 0.01).

The observation that non unique probe sets perform better than unique ones (figure 4c) is surprising because two probe sets each cross hybridize with a different set of genes may cause disagreement. However our results suggest the contrary – probes that were designed to recognize multiple genes, are much more abundant in the "good" pairs suggesting that the cross hybridization may have a smoothing affect that reduces the difference between the probes. In other words if the reason for the dis accordance between pairs is that each recognizes a unique transcript, less specific probe sets will mask this difference and thus will be more abundant among the "good" pairs of probe sets.

Our analysis suggests that in most cases (77%) pairs of informative probe sets report similar transcription levels. However, it should be noted that our decision to concentrate only on informative probe sets may influence those results. By excluding from the analysis the most problematic cases in which one probe set is informative and the other is not we elevated the percentage of cases with good correlation [see additional file 3]. These cases were omitted from the analysis because of the limited usefulness of the Pearson correlation on non informative probe sets (see methods). Data about the concordance between these pairs is included in additional file 1.

The analysis of the differences between the "good" and the "bad" pairs suggests that biological differences such as alternative splicing have a very limited contribution to the disagreement between probe sets. This conclusion was derived from the current knowledge of the genome sequence and of splicing variants (using fairly strict criteria). Updates of the genome sequence and advancements in the technology of measuring splice variants (such as the use of exon microarrays) may change this conclusion. Although those changes may affect the reasons for the disagreements between probe sets it will not change the definition of "good" and "bad" pairs. Only updating the probe set definition according to the new sequence information [18] may solve this problem but it is beyond the scope of the current study.

## Conclusion

An important internal control for microarray experiments is multiple array spots that are mapped to different regions of the same genes. In the ideal world these different probe sets should give similar measurements for expression of the gene. Our analysis tested whether this is true using four data sets that used the Affymetrix U133a platform. We found that in the majority of such cases both measurements agree and thus using the average value of the probe sets would be appropriate. However in a significant number of cases this approach is not suitable and our results can guide the user in deciding about each case. Further analysis is needed to define guidelines for choosing better regions in the gene that will represent the transcription level more accurately.

## Methods

### Data sets

For our analysis we downloaded the data of four large experiment series from the gene expression omnibus database [20]. We chose datasets with at least 29 experiments in order to have multiple measurements in each dataset. The GEO accession number, a short description of the experiments and the number of arrays used in each series are shown in table 1. All the expression values were log transformed.

### Pearson correlation

In order to define intra array repeats, we used Affymetrix annotation and defined 11,722 pairs of probe sets in which both members were mapped to the same gene. In order to compare the RNA levels reported by the two probes in each pair we calculated the Pearson correlation between the expression values (log transformed) reported by each probe set in all the experiments in each dataset. We also performed Spearman correlation and the results [see additional file 4] are very similar [see additional file 5].

Since we were interested only in the correlations between probe sets of active genes we looked only on probe sets that passed two filtering criteria – they got a present call (P) in at least 10% of the experiments in a dataset and they showed at least 2 fold changes in their expression level over the average in at least 10% of the experiments. This filtering resulted in a different number of informative pair of probe sets for each dataset (table 1).

Our decision to include in the analysis only cases were both probe sets in a pair are informative was based on the observation that the transcription values reported by non informative probe sets frequently reflect random noise fluctuations and thus the meaning of the correlation between those values and transcription values from informative probes is not clear. Nevertheless this decision introduced a bias in our analysis since cases in which the two probe sets have clear different characteristics were excluded. Calculating the correlation of the group of pairs in which only one of the probe sets was informative revealed that in all data sets the average correlation of this group of pairs was much closer to the average correlation between random pairs than to the average correlation of the pairs in which both probe sets were informative [see additional file 3].

### Statistical significance

In order to calculate the statistical significance of the correlation between the probe sets in each pair we transformed the values into Fisher Z values and compared those values to the average correlation values obtained for random pairs of informative probe sets using a Z test. The P values obtained for each data set were combined using the chi square test for combined q probabilities [21].

In order to define a P value threshold while accounting for the multiple hypothesis problem we used the FDR approach [22].

### Comparison between datasets

The fisher Z values obtained for each informative pair of probe sets in each dataset was used for comparing the results obtained in the different datasets. The comparison was done by calculating the Pearson correlation of those values. This procedure was repeated after shuffling the order of the pairs in order to obtain an estimation of the random correlation between the datasets.

### Error rate estimation

Transcription values were converted to ratios by dividing the value reported in each experiment by the probe set mean value in all experiments in a dataset. Cases with ratios greater than |2| were identified. This was done for individual probe sets and for the average values of two probe sets mapped to the same gene. Cases without full agreement were counted.

### Probe sets analysis

The accuracy of Affymetrix assignments of genes to probe sets was confirmed by comparing their assignments to those of the GeneAnnot database, which remapped Affymetrix probes to genes by performing blat searches with all individual probes forming the Affymetrix array [15]. This allowed us to divide the probe sets pairs into two categories – those which were mapped to the same gene using updated sequence information (85%) and those in which the current sequence information does not support affymetrix original annotation (15%). The number of transcripts mapped to each probe set was taken from the U133a annotation file provided by Affymetrix using the "RefSeq Transcript ID" column. Sequence homology between probe sets was determined by counting the number of identical probes shared by both probe sets in a pair. For the probe set uniqueness analysis we used the Affymetrix suffix. Affymetrix annotate each probe set with a suffix depicting whether it is unique to a single gene (_at), all its probes cross hybridize with multiple genes (s_at) or there is inconsistency between the probes forming the probe set in terms of cross hybridization with different genes (x_at) . We limited our analysis only to cases in which both probe sets in a pair have the same suffix.

The statistic significance of the difference between the distribution of "bad" and "good" probes was assessed with the chi square test or Fisher exact test.

## Authors' contributions

YE carried out most of the study and SF performed the Spearman correlation analysis. IS conceived the study, participated in its design and coordination and draft the manuscript. All authors read and approved the final manuscript.

## Supplementary Material

Additional File 1 Excel file containing information about all the pairs of probe sets in all the four datasets. The information provided includes the Pearson correlation values, the individual P values and the metrics used to filter non informative probe sets. Click here for file

Additional File 2 Excel file containing all the pairs of probe sets along with their combined P values. Click here for file

Additional File 3 Average Pearson correlation values between random pairs of probe sets (blue), pairs with only one informative probe set (red) and pairs in which both probe sets are informative (red). See methods for the definition of informative probe set. Click here for file

Additional File 4 Excel file containing information about the Spearman correlation values of all pairs of probe sets in all the four datasets. Click here for file

Additional File 5 Scatter plots showing the Pearson correlation values (X axis) against the Spearman correlation values (y axis) of all the informative pairs in the 4 data sets. Note that the spots are arranged along the diagonal with high R^2 ^values. The only dataset with spots that are not along the diagonal is GSE1133 in which ~5% of the spots deviate from the diagonal by >2 folds, the remaining spots gave R^2 ^= 0.93. Click here for file

## Figures and Tables

**Table 1:** 

Dataset accession number^1^	Description	Number of samples^2^	Number of informative pairs^3^
GSE1133	Gene atlas of human protein-encoding transcriptome from 79 tissues obtained from various sources.	158	4044
GSE473	Asthma and atopy – Investigation of CD4+ lymphocytes from patients with and without atopy, in combination with asthma	29	586
GSE974	Analysis of paired left ventricular apex samples harvested at implant of left ventricular assist device (LVAD) and at explant.	38	5148
GSE995	Expression profiling of HL-60 and acute promyelocytic leukemia cells treated with various drugs.	87	4695

^1 ^Of the gene expression omnibus database

^2 ^The number of experiments included in each dataset.

^3 ^The number of pairs included in the analysis in each dataset after removing non informative probe sets (see methods).
