# Supplementary material for: An analysis of intra array repeats: the good, the bad and the non informative
Source: BMC Genomics. 2006 Jun 5;7:136. doi: 10.1186/1471-2164-7-136 (PMC1501018; doi:10.1186/1471-2164-7-136)
Supplement: Additional File 3 — Average Pearson correlation values between random pairs of probe sets (blue), pairs with only one informative probe set (red) and pairs in which both probe sets are informative (red). See methods for the definition of informative probe set. [file 1471-2164-7-136-S3.PDF]

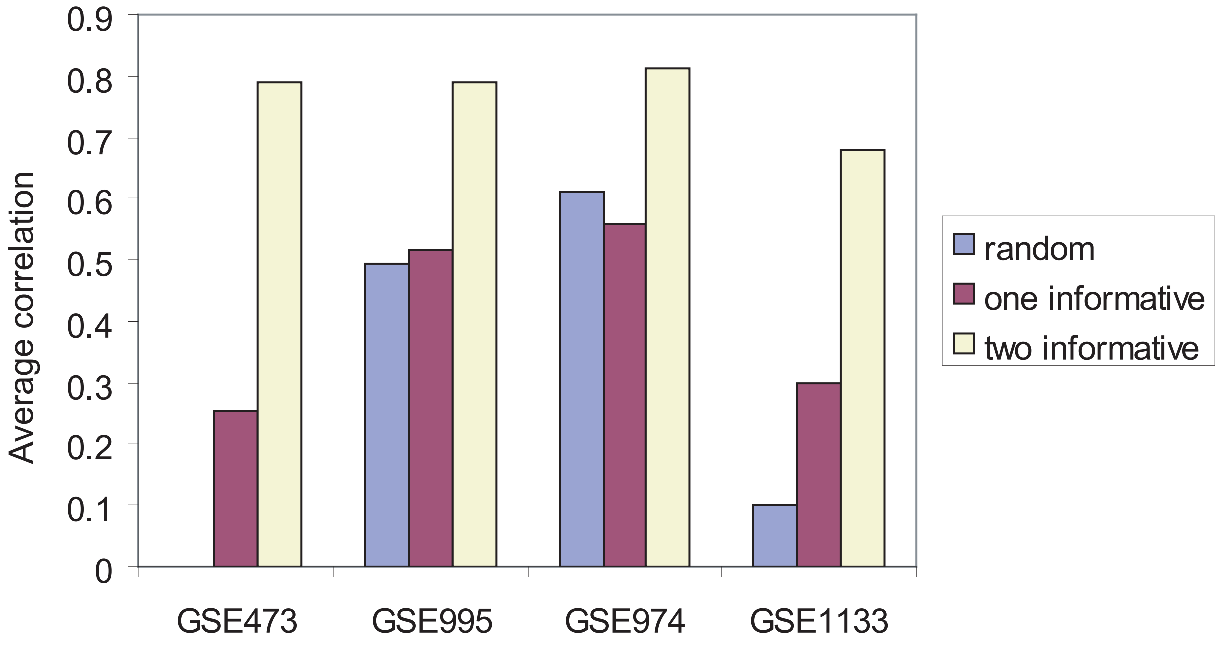

Supplementary figure 1
Average Pearson correlation values between random pairs of probe sets (blue), pairs with only one informative probe set (red) and pairs in which both probe sets are informative (red). see methods for the definition of informative probe set.
