# Supplementary figures and images for: An analysis of intra array repeats: the good, the bad and the non informative
Source: BMC Genomics. 2006 Jun 5;7:136. doi: 10.1186/1471-2164-7-136 (PMC1501018; doi:10.1186/1471-2164-7-136)

GSE995  $R^2=0.9078$

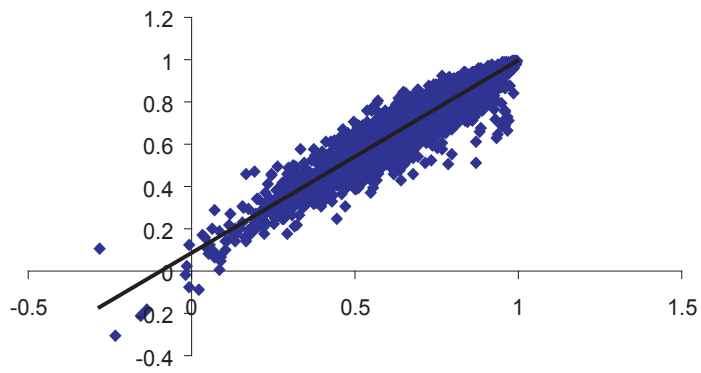

GSE473  $R^2=0.929$

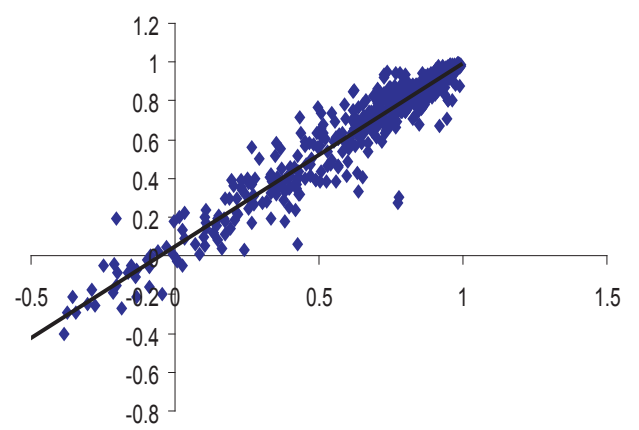

GSE974  $R^2=0.9075$

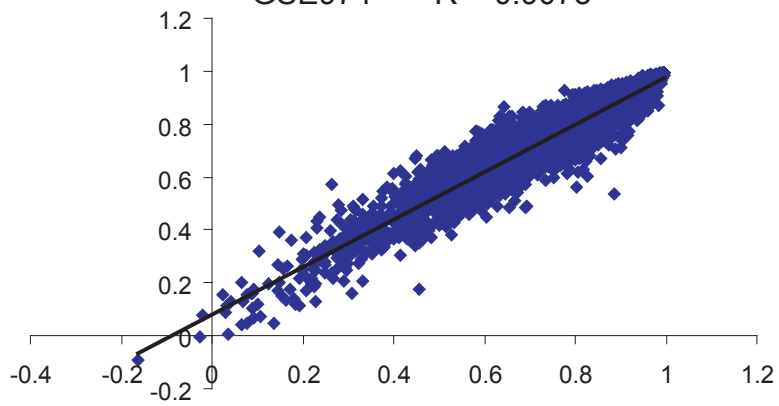

GSE1133  $R^2=0.7142$

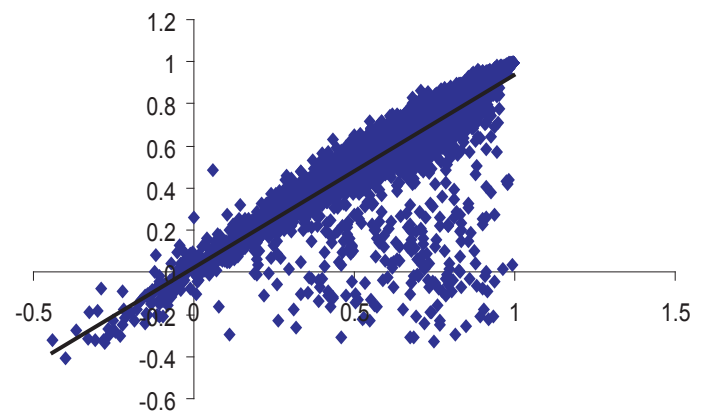

Supplement: Additional File 5 — Scatter plots showing the Pearson correlation values (X axis) against the Spearman correlation values (y axis) of all the informative pairs in the 4 data sets. Note that the spots are arranged along the diagonal with high R2 values. The only dataset with spots that are not along the diagonal is GSE1133 in which ~5% of the spots deviate from the diagonal by >2 folds, the remaining spots gave R2 = 0.93. [file 1471-2164-7-136-S5.pdf]
